# Malaria transmission in Libreville: results of a one year survey

**DOI:** 10.1186/1475-2875-11-40

**Published:** 2012-02-09

**Authors:** Jean-Romain Mourou, Thierry Coffinet, Fanny Jarjaval, Christelle Cotteaux, Eve Pradines, Lydie Godefroy, Maryvonne Kombila, Frédéric Pagès

**Affiliations:** 1 Département de Parasitologie-mycologie, Faculté de médecine, Université des Sciences de la Santé, B.P. 4009, Libreville, Gabon; 2 UMR 6236, Unité d'entomologie médicale, IRBA antenne Marseille, GSBDD Marseille Aubagne, 111 avenue de la corse BP 40026, 13568 Marseille Cedex 2, France

## Abstract

**Background:**

In Gabon, vector transmission has been poorly studied. Since the implementation of the Roll Back malaria recommendations, clinical studies have shown a decline in the burden of malaria in Libreville, the capital city of Gabon. To better understand the transmission dynamic in Libreville, an entomological survey was conducted in five districts of the city.

**Methods:**

Mosquitoes were sampled by human landing collection during 1 year in five districts of Libreville: Alibandeng, Beauséjour, Camp des Boys and Sotega. Mosquitoes were identified morphologically and by molecular methods. The *Plasmodium falciparum *circumsporozoïte indices were measured by ELISA, and the entomological inoculation rates (EIR) were calculated for all areas. Molecular assessments of pyrethroid knock down resistance (kdr) and of insensitive acetylcholinesterase resistance were conducted.

**Results:**

A total of 57,531 mosquitoes were caught during 341 person-nights (161 person-nights indoor and 180 person-nights outdoor) among which, 4,223 were *Anopheles gambiae s.l*. The average Human Biting Rate fell from 15.5 bites per person during the rainy season to 4.7 during the dry season. The *An. gambiae *complex population was composed of *An. gambiae s.s *molecular form S (99.5%), *Anopheles melas *(0.3%) and *An. gambiae s.s*. form M (0.2%). Thirty-three out of 4,223 *An. gambiae s.l*. were found to be infected by *P. falciparum *(CSP index = 0.78%). The annual EIR was estimated at 33.9 infected bites per person per year ranging from 13 in Alibandeng to 88 in Sotega. No insensitive AChE mutation was identified but both kdr-w and kdr-e mutations were present in *An. gambiae *molecular form S with a higher frequency of the kdr-w allele (76%) than the kdr-e allele (23.5%).

**Conclusion:**

Malaria transmission in Libreville occurred mainly during the rainy season but also during the dry season in the five districts. Transmission level is high and seems to be very heterogeneous in the town. Interestingly, the highest EIR was recorded in the most central and urbanized quarter and the lowest in a peripheral area. The decrease of transmission usually seen from peri-urban areas to urban centers is probably more dependent of the socio-economic level of a quarter than of its location in the city. Urban malaria control programmes need to consider the socio economic level of an area rather than the location in the city in order to determine the areas most favourable to malaria transmission.

## Background

Urbanization is increasing in Africa resulting in a change in the epidemiology of malaria [1]. One characteristic of African cities is the maintenance in urban areas of traditional rural practices, such as housing and food crops, that create favorable conditions for malaria transmission [2-4]. Marked intra-city variations in the burden of malaria exist due to differences in urbanization level, in equipment, in housing and in socio-economic level between districts [5]. Entomological inoculation rates can vary from 0 to 54 infective bites per man per year between Ouagadougou and Dar es Salaam and can double between the centre and periphery of a same city [6]. Currently, 40 African cities have more than one million inhabitants and in 2003, 39% of Africans lived in cities [7]. Keiser *et al. *estimated urban malaria morbidity to be between 25 and 100 million cases, amounting to between six and 28% of the total annual incidence [6]. According to the United Nations (UN) projections, by 2025, over 800 million people, about 54% of the continent's population, will live in urban areas (1). Thus, urban malaria has become an emerging public health problem in Africa and has become the subject of many studies to better understand its determinants, suggest preventive measures and appropriate control [8-15].

Gabon has a population, estimated at 1,534,381 inhabitants according to the Constitutional Court [16]. Despite it has one of the lowest population densities of any country in Africa, it is not spared by the demographic change that is affecting the majority of the continent. Due to a rural exodus, the majority (about 80%) of the population is urban or semi-urban [16]. Libreville alone has 579,577 inhabitants: 36.36% of the total population and 47.21% of the urban population [16]. Libreville is flanked by the Atlantic Ocean to the west and is irrigated by numerous rivers to the east. In Gabon, the burden of malaria has often been determined from clinical and laboratory data, by morbidity and mortality measurements, particularly at the Libreville Hospital Centre and in the Medical Research Unit of the Albert Schweitzer Hospital in Lambaréné [17,18]. Vector transmission has been poorly studied [19-21]. A preliminary study conducted between December 2006 and April 2007, during the rainy season in the French military base in Libreville (Camp de Gaulle), revealed the presence of *Anopheles gambiae s.l*. and showed a low transmission of four *Plasmodium falciparum*-infected bites per person per year [22]. This same study confirmed the high prevalence of molecular markers of resistance to pyrethroids described by Pinto *et al. *in 2006 and showed the presence of molecular markers of resistance to organophosphates and carbamates [23]. The military authorities have implemented an extensive anti-larval and anti-imago mosquito control in the camp, so the transmission level was relatively very low. However, this is probably not the reflect of the situation in the surroundings neighborhoods as it has been already shown for the non-anopheline mosquito vectors during the Chikungunya outbreak in 2007, when their density was 12 times higher outside the camp [24]. Reliable entomological data on the levels of transmission of malaria in different districts in Libreville is scant, despite the fact that it contains a third of the country's population.

Malaria in Libreville is a major public health problem as in other Central African capitals. In the 2000s, *P. falciparum *was responsible for 15% to 40% of medical care for fever in children under 11 years old [25]. During the same period, the prevalence of infection with *P. falciparum *and anemia were respectively 53.6% and 53% among pregnant women [26]. In 2005, artemisinin-based combination therapy (ACT) was adopted for uncomplicated malaria, long-lasting nets impregnated with pyrethroids (deltamethrin) were distributed to high risk groups (pregnant women and children under 5 years of age) and a strategy of intermittent preventive treatment with sulphadoxine-pyrimethamine for pregnant women (IPTp-SP) was made available in all health centers in the country [27]. Since then, clinical studies have shown a decline in the burden of malaria in Libreville both in pregnant women and in children [25,27]. Currently, data on parasite infection in febrile children aged 5 to 10 years old suggests a direct impact of this strategy on the level of exposure to malaria [25]. To better understand the transmission dynamics of malaria in Libreville, an entomological survey was conducted from December 2008 to January 2010 in five districts.

## Methods

### Study areas

**Figure 1:**
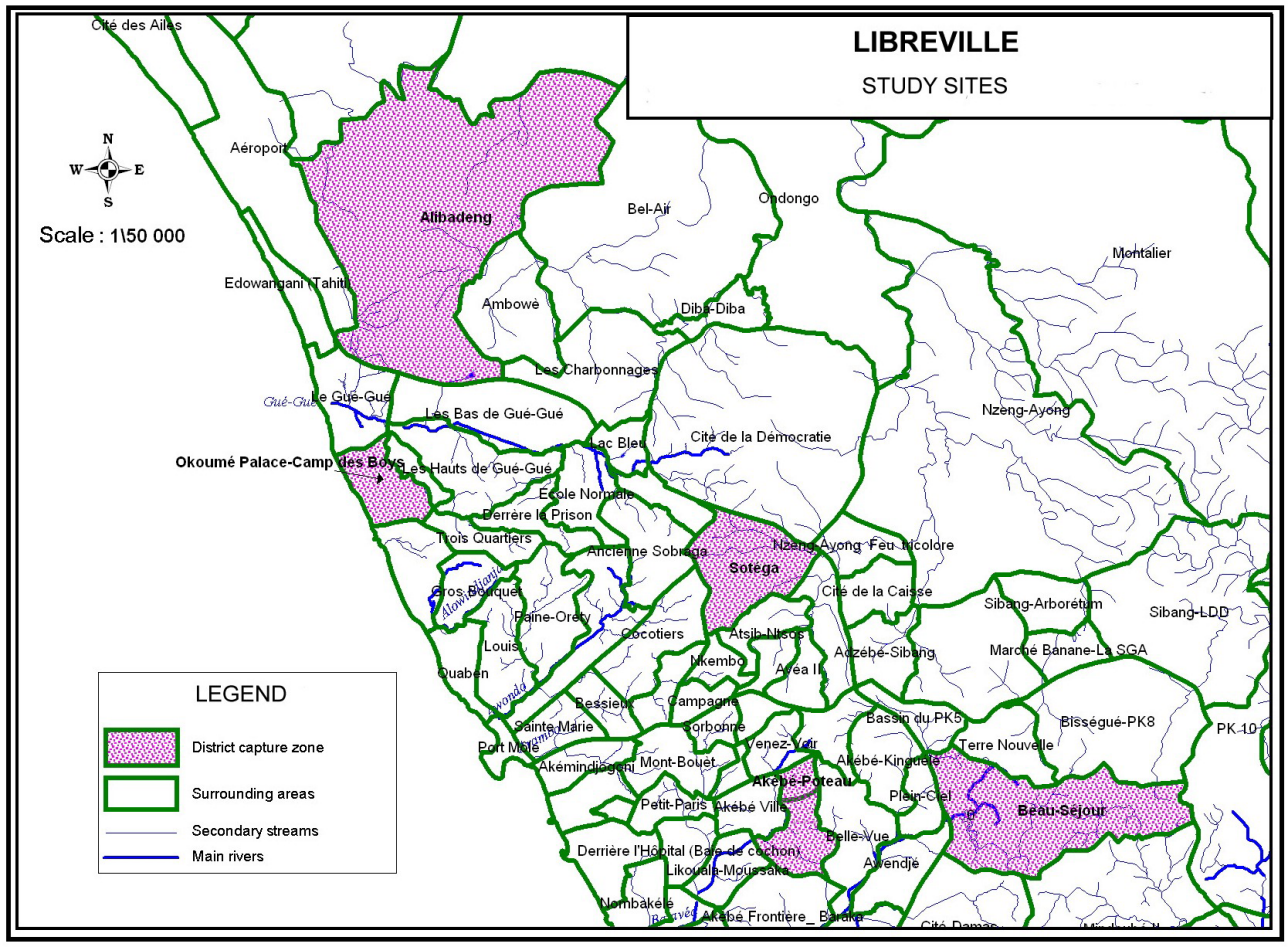
**Distribution of the study sites in Libreville**.

Libreville was initially partitioned in three areas considering the type of habitat, the location and the socio-economic level: a central zone with high population density and poor living conditions, an intermediate zone located on the coast where most neighborhoods are affluent and a suburban zone, separated from the central area by a highway, less densely populated, with dispersed houses (in between the intermediate and central zone). In each area, a district was randomly chosen in the list of Libreville districts: for the central zone, from July 2009, the district "Sotega" replacing Akébé-Poteau, followed from March to April 2009, for the intermediate zone area the district "Beau-séjour", for the peripheral zone the district "Alibandeng". Another district "Camp des Boys" neighboring the French military base "Camp de Gaulle" situated also in the peripheral area was added. The studied zones are presented and listed in Figure 1. As a specific survey of fevers by district was not possible for logistic reasons, we used data from the Department of Parasitology at the Libreville Medical School to determine the burden of malaria throughout the year. All patients presenting with fever from December 2008 to January 2010 had a blood smear taken.

### Climate and rainfall

**Figure 2:**
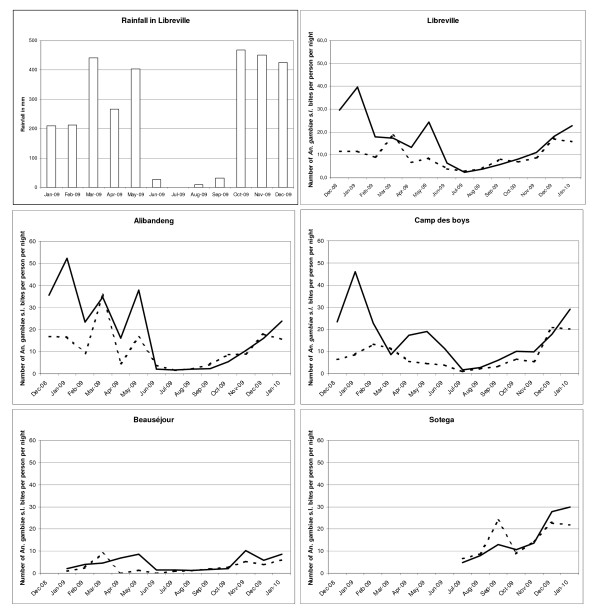
**Number of *An. gambiae s.l*. bites per person per night indoors (dotted line) and outdoors (black line) from December 2008 to January 2010 in four quarters of Libreville according to the 2009 rainfall in Libreville**.

Libreville is an equatorial climate characterized by a long rainy season lasting 8 months (interrupted by a short dry season) and a long dry season lasting 4 months. Average temperatures remain relatively constant throughout the course of the year, with average high temperatures at around 30°C. Rainfall data for 2009 were obtained courtesy of the General Directorate of Meteorology of Gabon and are presented in Figure 2. Annual rainfall was 2945.9 mm with a monthly average of 245.5 mm: maximum (468.7 mm) was seen in October and minimum (25.6 mm) in July. There were two seasons in 2009: a dry season from June to September, with a total rainfall of 67.8 mm (16.8 mm/month) and a rainy season from October to May, with a total rainfall of 2878.8 mm (359.85 mm/month). Despite a decline in rainfall during January-February (225.75 mm/month), it was not considered as a short dry season. For the purpose of the analysis, two main seasons were considered: a rainy season and a dry season.

### Processing of mosquito collections

Malaria vectors were sampled using both indoors and outdoors landing collections from December 2008 to January 2010. Collectors gave prior informed consent and received anti-malaria prophylaxis and yellow fever immunization. They were organized in teams of two for each collection point. Workers within a team were replaced every 2 h from 6:00 p.m. to 7:00 a.m. The teams rotated among the collection points on different nights to minimize sampling bias. In each district, four catching points were selected: two indoors and two outdoors. A capture session was performed every 2 weeks in each district. Occasional difficulties led to the addition or removal of some sessions or capture stations. In total, the capture effort was 341 man-nights: 15 for the district Akébé-Poteau (stopped tracking after 2 months), 60 for the district the "Sotéga", 97 for the district « Camp des Boys», 68 for the district « Beau séjour » and 101 for the district Alibandeng. Mosquitoes were recorded by location and hours of capture and were sorted by genera. Anopheline mosquitoes were identified morphologically following the Gillies and Coetzee keys [28]. *Culicinae *were identified morphologically following the Edwards keys [29]. All mosquitoes were stored individually in numbered vials with desiccant and preserved at -20°C until processing at the Medical Entomology Unit of the Institute for Biomedical research of the French Forces (IRBA), in Marseille (France).

### Laboratory mosquito processing

Heads and thoraces of anopheline females were tested by enzyme-linked immunosorbent assay (ELISA) for *P. falciparum *circumsporozoite protein (CSP) [30]. In each site, a random sample of females belonging to the *An. gambiae *complex, together with all CSP-positive anopheline, were identified by polymerase chain reaction (PCR) at the species and molecular form levels [31]. Molecular characterizations of the kdr and ace1 mutations were carried out on these mosquitoes as previously described [32,33].

### Data analysis

The human biting rate (HBR) was expressed as the number of female anopheline bites per human per night. Indoors, outdoors and global HBR were calculated. Values were averaged in order to calculate the HBR for the dry and the rainy season. The CSP index was calculated as the proportion of mosquitoes found to be positive for CSP. The entomological inoculation rate (EIR) was calculated as the product of the HBR and the CSP index of mosquitoes collected on humans. EIRs were calculated globally and by season (dry or rainy) for Libreville and for each district of study. The numbers of *An. gambiae s.l*. caught outdoors and indoors were standardized to calculate the endo-exophagic rates, then by district and by season. The CSP indices and the distribution of kdr alleles were compared using Chi^2 ^test or Fisher's exact test.

## Results

### Adult mosquito collection

A total of 57,531 mosquitoes were caught during 341 person-nights of collection on human bait (161 person-nights indoor and 180 person-nights outdoor). A total of 4,223 *An. gambiae s.l*. was collected (Table 1).

**Table 1:** Distribution by genus and species of adult mosquitoes collected on humans in the studied areas of Libreville

	Alibandeng		Camp des Boys		Beauséjour		Sotega		Akébé-Poteau		Total	
	101 person-nights		97 person-nights		68 person-nights		60 person-nights		15 person-nights		341 person-nights	
	
	N° of mosquitoes (%)	N° of bites per person per night	N° of mosquitoes (%)	N° of bites per person per night	N° of mosquitoes (%)	N° of bites per person per night	N° of mosquitoes (%)	N° of bites per person per night	N° of mosquitoes (%)	N° of bites per person per night	N° of mosquitoes (%)	N° of bites per person per night
*Cx. quinquefasciatus*	16862 (88.3)	166.9	14227 (91.3)	146.7	5001 (93.1)	73.5	12352 (91.7)	205.9	3737 (93.2)	249.1	52179 (90.7)	153.0

*Mansonia sp*	339 (1.8)	3.4	73 (0.5)	0.75	41 (0.8)	0.6	152 (1.1)	2.5	14 (0.3)	0.9	619 (1.1)	1.8

***An. gambiae s.l***.	**1611 (8.4)**	**16**	**1236 (7.9)**	**12.7**	**275 (5.1)**	**4.0**	**962 (7.1)**	**16.0**	**139 (3.5)**	**9.3**	**4223 (7.3)**	**12,4**

*Ae. aegypti*	130 (0.7)	1.3	30 (0.2)	0.3	21 (0.4)	0.3	#	0.0	110 (2.7)	7.3	291 (0.5)	0.9

*Ae. albopictus*	146 (0.8)	1.4	22 (0.1)	0.2	32 (0.6)	0.5	8 (0.1)	0.1	11 (0.3)	0.7	219 (0.4)	0.6

**Total**	19 088	189	15 588	160.7	5 370	79.0	13 474	224.6	4011	267.4	57 531	168.7

### Biting rates and biting behaviours of *An. gambiae s.l*

**Figure 3:**
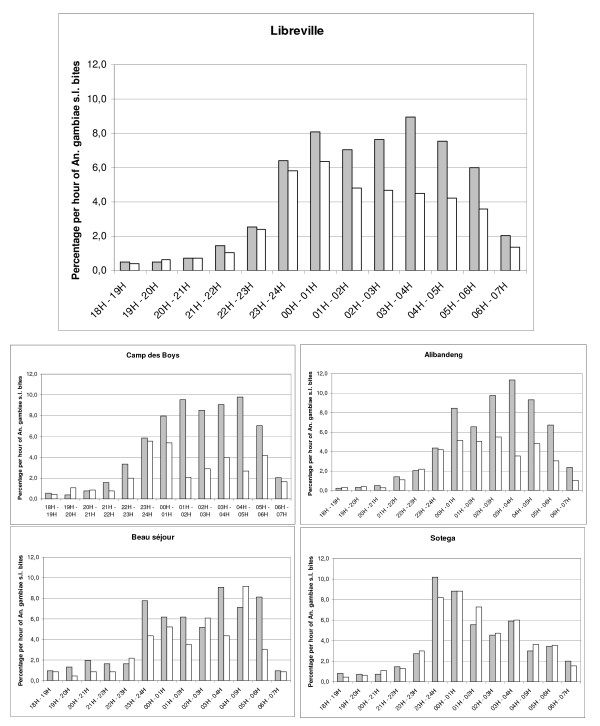
**Hourly distribution of *An. gambiae s.l*. bites indoors (white bars) and outdoors (gray bars) in Libreville from December 2008 to January 2010**.

Overall, the peak biting time was between 11:00 p.m. and 5:00 a.m. (Figure 3) with 89.1% of the bites occurring after 11:00 p.m. Human landing catches gave an average biting rate of 12.4 *An. gambiae s.l*. bites per person per night (14.5 outdoors; 9.9 indoors) for all Libreville. There were differences between the study areas: the average biting rate for *An. gambiae s.l*. ranged from four in Beauséjour (4.6 outdoors; 3.3 indoors) to 16 in Alibandeng (20.1 outdoors; 11.6 indoors). For the others districts (Akébé-Poteau, Camp des Boys and Sotega), the average biting rates were respectively of 9.3 (7.7 outdoors; 11 indoors), 12.7 (16.7 outdoors; 8.4 indoors) and 16 (15.8 outdoors; 16.2 indoors). The biting activity in Libreville and in its districts according to rainfall from December 2008 to January 2010 is shown in Figure 2. The aggressiveness of *An. gambiae s.l*. differed also by season. During the rainy season, the average biting rate for *An. gambiae s.l*. was 15.5 bites per person per night, with a peak of 40 bites per person per night; this fell to 4.7 bites per person per night during the dry season. The differences of *An. gambiae s.l*. biting rates between districts persisted from the rainy to the dry season. For Alibandeng, Camp des Boys, Beauséjour and Sotega, the average biting rate fell respectively from 20.6 bites per person per night to 2.5, from 15.8 to 4.3, from 5.3 to 1.5 and from 19.5 to 10.8. The density of the *An. gambiae s.l*. populations in the different areas was clearly linked to the level of rainfall. Each peak or decrease of rainfall was responsible for an increase or decrease in the population of *An. gambiae s.l*., respectively, the following month.

**Figure 4:**
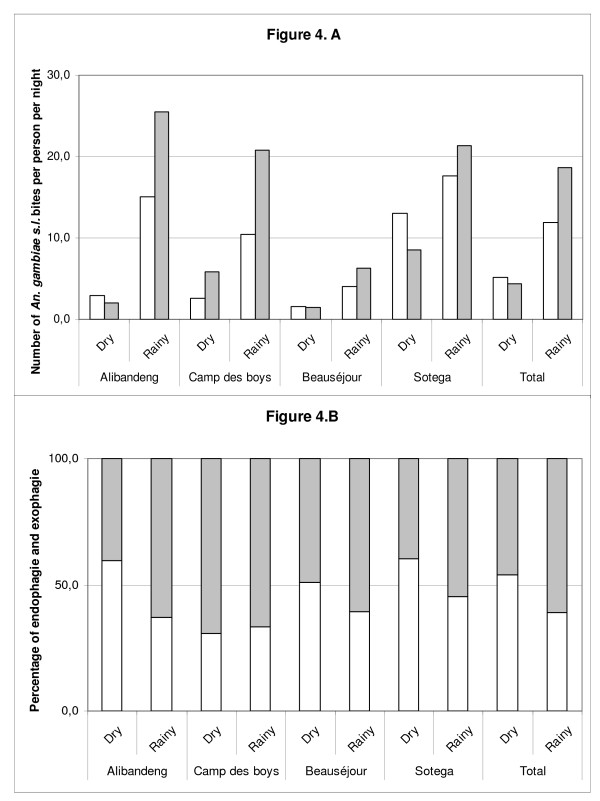
**A Number of *An. gambiae s.l*. bites per person per night indoors (white bars) and outdoors (gray bars) according to the season in four quarters of Libreville**. **B**. Variations of the endo-exophagic behaviours of *An. gambiae s.l*. in four quarters of Libreville according to the season.

Among the 4,223 *An. gambiae s.l*. caught, 2,622 (62.1%) were caught outdoors, indicating that this species was globally more exophagic in Libreville. This was confirmed in three districts: Alibandeng (64.8%), Camp des Boys (68.8%) and Beauséjour (65.1%). This was significantly different from the two other districts: Sotega (50.6%) and Akébé-Poteau (44.6%), where *An. gambiae s.l*. seemed to be more endophagic (Chi^2 ^test, p < 10^-7^). Data on location (indoor vs outdoor) and by season of *An. gambiae s.l*. are presented in Figure 4, by district. *An. gambiae s.l*. seemed to modify its host seeking behaviour between the dry and the rainy season and was more endophagic during the dry season (RR = 1.71[1.45-2.02]), however, when analysed at the district level, this phenomenon is only seen in two districts: Alibandeng (RR = 2.39[1.44-3.99]) and Sotega (RR = 1.47[1.19-1.82]).

### Molecular identification *of An. gambiae s.l*

All specimens caught during the dry season along with a random sample of specimens caught during the rainy season were identified by PCR. Among the 1,006 specimens tested by PCR, the *An. gambiae complex *population was composed of 1,001 *An. gambiae s.s*. molecular form S (99.5%), 3 *An. melas *(0.3%) and 2 *An. gambiae s.s*. form M (0.2%). *Anopheles melas *and *An. gambiae *molecular form M were caught only during the rainy season: *An. gambiae *molecular form M was present only in Alibandeng and *An. melas *specimens were caught in Camp des Boys, Beauséjour and Sotega.

### CSP and EIR

All 4,185 *An. gambiae s.l*. collected by human landing catches were processed by ELISA for *P. falciparum *antigen detection (467 from the dry season and 3,718 from the rainy season) and 33, all *An. gambiae s.s*. molecular form S, were found to be positive. CSP indexes and EIRs are presented in Table 2. As no statistically significant differences existed between the CSP indexes for the rainy and the dry season, an annual CSP index was calculated globally and for the four districts studied during both rainy and dry seasons, thus allowing the calculation of annual EIRs globally and by district. When the annual CSP indexes by district were compared, the annual CSP index in Alibandeng was statistically different from the others quarters but no difference was seen between Camp des Boys, Beauséjour and Sotega. An annual averaged CSP index and their respective annual EIRs were calculated for these three areas. The annual CSP index in Libreville was 0.78% [0.54-1.09]. The annual EIR was estimated at 33.9 infected bites per person per year (23.4 min and 47.3 max). Most malaria transmission occurred during the rainy season (87% of infected bites per person per year) but malaria transmission also occurred during the dry season from June to September. The level of transmission varied according to district: an inhabitant in Sotega receives about 88 infected bites per year, whereas an inhabitant in Alibandeng receives about 13 infected bites during the same period.

**Table 2:** Mean daily human biting rate (HBR) during the dry and the rainy season; dry, rainy and annual CSP indices; dry, rainy and annual entomological inoculation rates (EIR) of An. gambiae s.l. by sites in Libreville: data collected from December 2008 to January 2010

	Dry season					Rainy season					All year				
	
	Total collected	HBR	Tested for CSP	CSP rate [95% C.I]	Dry season EIR	Total collected	HBR	Tested for CSP	CSP rate [95% C.I]	Rainy season EIR	Dry CSP/Rainy CSP	Annual CSP	Annual EIR	Annual averaged CSP*	Annual averaged EIR*
Alibandeng	65	2.5	65	0 [0.0; 5.5]	0.0	1546	20.6	1526	0.26 [0.07;0.67]	2.5	NS	0.25 [0.06;0.64]	13.3	-	-

Camp des Boys	109	4.3	108	0.93 [0.02;5.05]	4.7	1127	15.8	1113	0.89 [0.43;1.64]	34.5	NS	0.9 [0.45;1.60]	39.2	1.11	48.4

Beauséjour	35	1.5	35	0 [0.0;10.0]	0.0	240	5.3	239	0.84 [0.10;2.89]	9.3	NS	0.72 [0.08;2.61]	10.6	1.11	16.3

Sotega	260	10.8	259	1.16 [0.23;3.34]	19.2	702	19.5	701	1.57 [0.78;5.1]	68.7	NS	1.45 [0.79;2.43]	87.9	1.11	67.3

Akébé-Poteau	-	-	-	-	-	139	9.2	139	1.43 [0.17;5.1]	31.9	-	1.43 [0.17;5.1]	-	-	-

Total	469	4.7	467	0.85 [0.23;2.17]	4.5	3754	15.5	3718	0.78 [0.52;1.11]	29.4	NS	0.78 [0.54;1.09]	33.9	-	-

*Annual CSP indexes of the four areas studied during the two seasons have been compared using Chi^2 ^test. Only the Annual CSP index of Alibandeng was statistically different of the others. An annual averaged CSP index has been calculated for the three remaining areas.

### Kdr and ace1 mutation frequencies in *An. gambiae s.l*

All CSP-positive mosquitoes per site and a random sample of at least 100 PCR-identified mosquitoes were tested for the kdr and ace1 mutations. The Ace 1 mutation was not identified in any of the 783 members of the *An. gambiae *complex collected in the five areas of Libreville (163 in Alibandeng, 209 in Camp des Boys, 121 in Beauséjour, 186 in Sotega and 104 in Akébé-Poteau). A total of 1020 *An. gambiae *molecular form S were tested for the detection of pyrethroid knock-down resistance. Both kdr-e and kdr-w resistance alleles were present with a higher frequency for kdr-w allele (76.0%) than kdr-e allele (23.5%). The genotypic and the allelic frequencies are shown in Table 3. The comparison of the allelic frequencies showed no difference between districts.

**Table 3:** Distribution of the genotypic and allelic frequencies of the kdr loci of *An.gambiae s.s*. molecular form S in the studied areas of Libreville

		Genotypic frequencies					Allelic frequencies				
		
	N° of specimens	ReS	ReRe	ReRw	RwRw	RwS	SS	Re	Rw	S	p*
Alibandeng	240	1 (0.4)	13 (5.4)	80 (33.3)	146 (60.9)	-	-	22.3	77.5	0.2	

Camp des boys	310	-	18 (5.8)	120 (38.7)	168 (54.2)	2 (0.6)	2 (0.6)	25.1	73.9	1	

Beauéjour	138	-	7 (5.1)	48 (34.8)	82 (54.4)	-	1 (0.7)	22.5	76.8	0.7	NS

Sotega	203	-	15 (7.4)	63 (31.0)	124 (61.1)	-	1 (0.5)	22.9	76.6	0.5	

Akébé Poteau	129	-	8 (6.2)	44 (34.1)	77 (59.7)	-	-	23.3	76.7	0	

Libreville-Total	1020	1 (0.1)	61 (6.0)	355 (34.8)	597 (58.5)	2 (0.2)	4 (0.4)	23.5	76.0	0.5	

p* the allelic frequencies of the kdr loci are not different between the different districts of Libreville

### Malaria in patients consulting for fever

**Figure 5:**
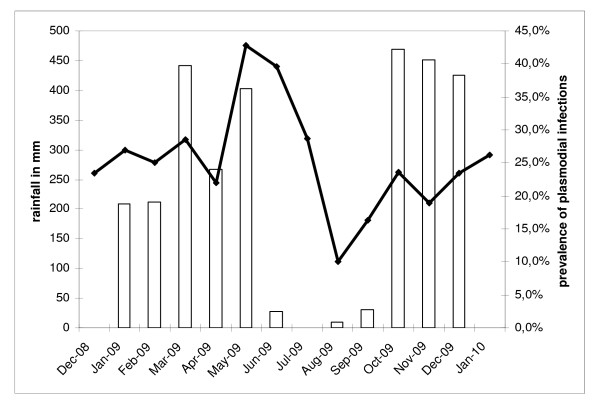
**Evolution by month of the rainfall (white bars) and of the prevalence of plasmodial infections in patients consulting for fever (black line) in Libreville from December 2008 to January 2010**.

From December 2008 to January 2010, 1,730 children (under 15 years old) of both sexes consulted for fever: 419 of them (24.2%) were infected by *P. falciparum*. The ratio of infected patients is described in Figure 5. The proportion of infected patients is higher in the last months of the rainy season and dropped during the dry season only to increase again with the start of the rainy season.

## Discussion

The nocturnal mosquito biting rate was intense essentially due to *Culex quinquefasciatus *in both areas of Libreville but varied according to the location in the city. The *Cx. quinquefasciatus *aggressiveness ranged from 73 bites per night in Beauséjour to 250 bites per night in Akébé-Poteau. The densities of *Cx. quinquefasciatus *were higher in the centre of the city in the most urbanized areas, suggesting difficulties in waste management, and were lower in the peripheral areas of Alibandeng and Camp des Boys. *Stegomya spp*. population densities were low during the survey, but night captures are not appropriate for such diurnal species. The abundance of *Aedes aegypti *and *Aedes albopictus *seemed to vary according to the districts signifying that the risk of chikungunya or dengue transmission is probably not homogenous throughout Libreville. In Akébé-Poteau, a more urbanized area with less vegetation, *Ae. aegypti *was more present than *Ae. albopictus*. In the others districts, where gardens and other green areas are more important the two species were caught with equal frequency.

Three members of the *An. gambiae *complex were present in Libreville: *An. melas, An. gambiae s.s*. molecular form M and *An. gambiae s.s*. molecular form S. The presence of *An. melas *and *An. gambiae s.s*. molecular form M was recorded for the first time in Libreville. Only *An. gambiae s.s*. molecular form S, that represented more than 99% of the anopheline population, was involved in malaria transmission, which is contrary to what is observed in Port Gentil, the second main city of the country, where *An. melas *is an important malaria vector [22]. The level of transmission varied between the districts and the annual EIR ranged from 13 to 87 *P. falciparum *infected bites per person per year. Differences between districts can be explained in part by the variations in *An. gambiae s.s*. molecular form S biting rates for three of the districts however, in Alibandeng, where the annual aggressiveness was one of the highest, the lower level of transmission is possibly due to a low CSP index and to a higher exophagic behaviour of *An. gambiae s.l*..

A meta-analysis of studies of malaria transmission in sub-Saharan Africa found a linear negative relationship between the level of malaria transmission and the level of urbanization: transmission decreased from rural to peri-urban areas and from peri-urban areas to urban centre [34] and these findings have been confirmed in the field [35]. In Libreville, the situation appears different as the highest EIR was in the most central and urbanized area and the lowest in a peripheral area. In urban settings, malaria risk heterogeneity is due to diversity in degrees and types of urbanization, density of human population, quality of water and waste management, vector control measures, household factors and access to health care [7,14,34,36,37] or human migration patterns which could import parasites from rural areas [38-40]. The occurrence of malaria in African cities has been linked to agricultural practices [5,41-45], distance from breeding sites [46-50] and vegetation cover [50]. In Libreville, some of these factors could explain the differences between the intermediate area (Beauséjour), the peripheral area (Alibandeng) and the central zone (Sotega) and the apparent inversion of the usual gradient of transmission from peri-urban areas to urban centre. Households with low socio-economic status and poor housing conditions have already been identified as risk factors for urban malaria. In the central area, districts like Sotega which have slum-like conditions characterized by a high density of population, a low socio-economic level and difficulties in waste management, as seen by the high biting rate of *Cx quinquefasciatus*. Contrary to the usual scheme described in most of African cities, the peripheral and intermediate areas are more affluent, less densely populated and inhabitants have a higher socio economic status than those of the central area of Sotega. The gradient of transmission described from peri-urbans areas to urban centre is probably more dependent of the socio-economic status of the area than of the location within a city. Urban malaria control programmes thus need to consider living conditions in a given area rather than the location in a city to determine areas favourable to malaria transmission. In 2007, a preliminary study conducted in the French military camp showed a low transmission of malaria in Libreville [22]. However, the authors identified two limitations: first, a single study in one area is not sufficient to assess the global level of transmission and second, that the vector control programme implemented in the camp all year around could have minimized the level of malaria transmission. This current study included the district Camp des Boys due to its proximity to the French military camp of Libreville, and found that the level of transmission was ten fold higher with a measured annual EIR of 39.2 infected bites per person per year vs. the estimated annual EIR of 3.7 infected bites per person per year in the French military camp. It is the first study showing the effectiveness of the vector control programme implemented against malaria vectors in the French camps in Africa [24].

This study also showed that most of malaria transmission occurred during the rainy season. As the CSP indexes were not statistically different between the rainy and the dry seasons, this variation is due to the climatic conditions that are more favourable to *An. gambiae s.l*. populations during the rainy period. Rainfalls provide larvae breeding sites, allowing an increase in the density of the populations and a higher level of hygrometry increases the longevity of the populations and therefore their vector capacity. The predominant presence of *S *form is further evidence of the importance of rain-dependent temporary breeding sites in Libreville [51].

The evolution of the proportion of *P. falciparum *infected persons in patients consulting for fever is proof of the link between malaria transmission and rainfall. However, many people spend their summer holidays in the inlands, and the increase of malaria transmission seen in October after the return of people could be explained by the importation of *P. falciparum *to the city by returning vacationers and students [38-40]. Preliminary work conducted in 2007 showed that the aggressiveness of *An. gambiae s.l*. was linked to rainfall; this study showed that malaria transmission in Libreville is clearly linked to rainfall. Malaria transmission primarily occurred during the rainy season, but transmission persisted during the dry season, though it was six times lower (4.5 infected bites per person during the dry season vs. 29.5 infected bites per person during the rainy season).

As *An. gambiae s.l*. have been caught both indoors and outdoors, malaria transmission can occur both indoors and outdoors. During the dry season, *An. gambiae s.l*. seemed to modify its host-seeking behaviour and to penetrate more easily in the houses. This modification of the biting behaviour could participate to the maintenance of malaria transmission during the dry season. Considering the four districts followed during the two seasons, *An. gambiae s.l*. was endophagic in one of them (Sotega). Interestingly, malaria transmission was higher in this district during the dry season.

The human night biting pattern supports the efficacy of impregnated nets in malaria prevention. Nevertheless, some biting activity takes place at the end of night (14% of bites from 5:00 to 7:00 a.m.) when people don't use any protection. As recommended by WHO, repellents are used by French forces stationed in sub-Saharan countries to complete protection, outside of the period of use of mosquito nets even in the early morning [5,52-54]. A successful trial in Amazonia has showed the efficiency of this strategy using a "natural" locally produced repellent [55]. However, plant extracts have limited protection, for a short duration "natural" repellents traditionally used in Africa can be proposed [56-58].

As previously described, in Libreville, both kdr-w and kdr-e mutations in *An. gambiae *molecular form S were present [23,59] with a higher frequency of the kdr-w allele (76%) than the kdr-e allele (23.5%) as reported in neighbouring countries [59-62]. As those mutations have been tightly linked with resistant phenotypes, the effectiveness of the current distribution programme of pyrethroid-impregnated bed nets in Libreville has to be assessed however none consensus has been reached on the impact of kdr mutations on the efficacy of ITNs [63-69]. No insensitive AChE mutations were found in the five districts, suggesting that the molecular resistance to organophosphates and carbamates insecticides in *An.gambiae s.s*. form S has not infiltrated Libreville and that these compounds are still a viable alternative.

## Conclusions

Malaria transmission in Libreville remains high and seems to be very heterogeneous throughout the city. New studies, such as entomologic surveys and human surveys, are needed to better understand the factors of this heterogeneity. As an uncontrolled use of carbamates or organophosphates could probably lead to select multi-resistant specimens, the use of molecular markers has to be developed in Gabon as a routine tool for malaria control programme deciders, as well as the use of standardized bio-essays -to take into account the possible involvement of additional resistance mechanisms-, as it has begun in the neighbouring countries [70-72].

## Competing interests

The authors declare that they have no competing interests.

## Authors' contributions

JRM was responsible for the supervision of data collection, analysis, interpretation and production of the final manuscript and revisions. TC contributed to the supervision of data collection, the data analysis, and interpretation. FJ contributed to the supervision of data collection, to the data analysis. CC contributed to the data analysis and production of final manuscript. EP contributed to the data analysis. LG contributed to the data analysis. MK contributed to overall scientific management, analysis, interpretation and preparation of the final manuscript and revisions. FP was responsible for overall scientific management, analysis, interpretation and preparation of the final manuscript and revisions. All authors read and approved the final manuscript.
